# Germline polymorphisms in *SIPA1 *are associated with metastasis and other indicators of poor prognosis in breast cancer

**DOI:** 10.1186/bcr1389

**Published:** 2006-03-21

**Authors:** Nigel PS Crawford, Argyrios Ziogas, David J Peel, James Hess, Hoda Anton-Culver, Kent W Hunter

**Affiliations:** 1 Laboratory of Population Genetics, National Cancer Institute/National Institutes of Health, Bethesda, Maryland, USA; 2 Epidemiology Division, Department of Medicine, University of California, Irvine, California, USA

## Abstract

**Introduction:**

There is growing evidence that heritable genetic variation modulates metastatic efficiency. Our previous work using a mouse mammary tumor model has shown that metastatic efficiency is modulated by the GTPase-activating protein encoded by *Sipa1 *('signal-induced proliferation-associated gene 1'). The aim of this study was to determine whether single nucleotide polymorphisms (SNPs) within the human *SIPA1 *gene are associated with metastasis and other disease characteristics in breast cancer.

**Method:**

The study population (*n *= 300) consisted of randomly selected non-Hispanic Caucasian breast cancer patients identified from a larger population-based series. Genomic DNA was extracted from peripheral leukocytes. Three previously described SNPs within *SIPA1 *(one within the promoter [-313G>A] and two exonic [545C>T and 2760G>A]) were characterized using SNP-specific PCR.

**Results:**

The variant 2760G>A and the -313G>A allele were associated with lymph node involvement (*P *= 0.0062 and *P *= 0.0083, respectively), and the variant 545C>T was associated with estrogen receptor negative tumors (*P *= 0.0012) and with progesterone negative tumors (*P *= 0.0339). Associations were identified between haplotypes defined by the three SNPs and disease progression. Haplotype 3 defined by variants -313G>A and 2760G>A was associated with positive lymph node involvement (*P *= 0.0051), and haplotype 4 defined by variant 545C>T was associated with estrogen receptor and progesterone receptor negative status (*P *= 0.0053 and *P *= 0.0199, respectively).

**Conclusion:**

Our findings imply that *SIPA1 *germline polymorphisms are associated with aggressive disease behavior in the cohort examined. If these results hold true in other populations, then knowledge of *SIPA1 *SNP genotypes could potentially enhance current staging protocols.

## Introduction

Breast cancer is a major public health concern among Western female populations. In 2004 breast cancer was the most common form of malignancy diagnosed in females in the USA and the second leading cause of cancer mortality in women [1]. The great majority of these deaths are related to complications caused by metastatic disease, and in spite of therapeutic advances metastatic breast cancer is currently incurable [2]. It has been estimated that 24–30% of women with node-negative disease and at least 50–60% of those with node-positive disease at diagnosis will relapse [3]. Furthermore, approximately 6–10% of breast cancer patients present with clinical evidence of metastasis, and the median survival of those with metastatic disease is estimated to be 2–4 years [3].

The impact of metastatic disease on prognosis underscores the importance of improving upon currently available means of diagnosing and treating advanced breast cancer. One potentially powerful approach would be to identify early those patients who are at high risk for disseminated disease and then administer tailored treatment to reduce or eliminate the emergence of secondary lesions. As our knowledge of both the genetic and molecular mechanisms underpinning metastasis improves, it is becoming apparent that use of simple, reliable and robust assays that rely upon stable, constitutional fingerprints, including germline DNA polymorphisms, may provide an alternative means of identifying patients at risk for advanced disease. Recent studies have demonstrated that low penetrance germline polymorphisms influence overall susceptibility to breast cancer (for review, see the report by Houlston and Peto [4]) as well as the likelihood of tumor progression and/or recurrence (for review, see the report by Foulkes and coworkers [5]). Probably the best documented genes with respect to the latter are *BRCA1 *and *BRCA2*, with specific germline mutations being demonstrated to be associated with various indicators of poor outcome [6-9].

Previously, our laboratory provided evidence that the host genetic background upon which a tumor arises can significantly alter metastatic efficiency. This was demonstrated by a breeding scheme in which the highly metastatic polyoma middle T transgenic mouse mammary tumor model was bred to a variety of inbred strains and the metastatic capacity of the tumors in each of the F_1 _hybrid populations determined [10]. Wide variations in metastatic efficiency for each of the different F_1 _progeny were observed following mammary tumor development [10], and because all of the tumors were induced by the same transgenic event – namely the activation of the polyoma middle T antigen [11] – it was concluded that the observed difference in metastatic efficiency in F_1 _progeny was a consequence of germline genetic variation between each of the different inbred strains.

Quantitative trait mapping experiments revealed the presence of a metastasis efficiency modifier locus linked to the proximal end of mouse chromosome 19 [12]. Subsequent evaluation of this locus identified a gene called *Sipa1 *('signal-induced proliferation-associated gene 1'; also known as *Spa1*) as a likely candidate for this modifier locus. Sequence analysis of *Sipa1 *revealed an alanine to threonine mutation in the PDZ domain in mice with high metastatic potential (FVB strain, AKR strain) when compared with low metastatic phenotype mice (NZB strain, DBA strain) [13]. Computer modeling of the wild-type and mutant forms of *Sipa1 *indicated that this mutation occurs on the open face of an α-helix, and *in vitro *analysis demonstrated that the polymorphism functionally impacts on protein-protein interactions. Furthermore, these data indicated that the metastatic capacity of mammary tumors in spontaneous metastasis assays was significantly altered by modulation of *Sipa1 *expression. Metastatic capacity was increased by *Sipa1 *upregulation as a consequence of ectopic gene expression [13], and a decrease in metastasis was seen when *Sipa1 *expression was reduced by RNA interference utilizing *Sipa1*-specific short-hairpin RNAs.

The aim of this study is to determine whether *SIPA1 *(MIM# 602180) plays a similar role in the modulation of metastatic potential in human breast cancer populations. We hypothesized that polymorphisms or haplotypes within key regulatory or coding elements of the human gene encoding *SIPA1*, located on chromosome 11q13.3, are positively correlated with the presence of metastases in breast cancer patients. To test this hypothesis, we designed a case-only association study in which a number of single nucleotide polymorphisms (SNPs) in *SIPA1 *were characterized in patients with metastatic or nonmetastatic breast cancer.

## Materials and methods

### Recruitment of patients

This study was approved by the institutional review boards of the US National Institutes of Health and the University of California (Irvine, CA, USA). Written informed consent was obtained from all patients. The patients included in this study are a subgroup of 300 women with breast cancer selected from a larger population-based population of incident breast cancer patients. Breast cancer probands diagnosed between 1 March 1994 and 28 February 1995 were identified through the population-based cancer registry of the Cancer Surveillance Program of Orange County. Description of the Program and details of data collection methods were reported previously [14]. The overall goal of the parent study was to provide a means to identify a large fraction of the population that is at genetically high risk for breast or ovarian cancer, and to determine the significance of inherited cancer-predisposing genes as risk factors for cancer development in the population.

We report here data on 300 breast cancer patients (all non-Hispanic white women) selected from among the patients included in the parent study as follows: approximately 51% of patients with regional or metastatic breast cancer (154 cases); and randomly selected patients with localized disease (146 cases). Regional disease indicates direct extension of the primary tumor to the skin, muscle, chest wall, lymph nodes, or a combination of the above; metastatic disease indicates all other forms of involvement beyond regional disease. Pathologic diagnoses were obtained at the time of initial presentation in all cases, and the methods used for data collection were standardized. The protocol included initial contact with patients' physician followed by a mail-based invitation and description of the study. After patients had consented to participate in the study, family history and epidemiologic risk factors were assessed by telephone interview. In addition, data on tumor characteristics and stage at diagnosis were collected from the cancer registry. These data were based on pathology reports and medical records of the cancer patients. Blood samples were collected from all cancer patients, DNA was extracted from each sample, and aliquots were sent to the NCI (IRB #HS-2004-3832).

### Selection of single nucleotide polymorphisms

We characterized three SNPs within the regulatory or coding regions of *SIPA1*. SNPs were chosen on the basis of their genomic location from the NCBI SNP database [15]. One polymorphism, -313G>A (NCBI SNP designation rs931127) is located 313 base pairs upstream of the 5'-untranslated region of *SIPA1 *and is considered to be within the promoter region of the gene. The remaining two SNPs are located within coding regions, one within exon 1 (545C>T [F182S]; rs3741378) and one within exon 12 (2760G>A [A920A]; rs746429).

### Single nucleotide polymorphism genotyping

*SIPA1 *polymorphisms were characterized using SNP-specific PCR. The PCR primers were designed using Vector NTI 9.0 software (Invitrogen, Carlsbad, CA, USA), in accordance with parameters described elsewhere [16]. Each probe was labeled with a reporter dye (either VIC^® ^[a proprietary fluorescent dye produced by Applied Biosystems, Foster City, CA, USA] or FAM [5-(&6)-carboxyfluorescein]) specific for wild-type and variant alleles of each of three *SIPA1 *SNPs, respectively. Sequences of PCR primers and fluorogenic probes are given in Table 1.

Reaction mixtures consisted of 300 nmol/l of each oligonucleotide primer, 100 nmol/l fluorogenic probes, 8 ng template DNA, and 2× TaqMan Universal PCR Master Mix (Applied Biosystems) in a total volume of 10 μl. The amplification reactions were performed in a MJ Research DNA Engine thermocycler (Bio-Rad, Hercules, CA, USA) with two initial hold steps (50°C for 2 minutes, followed by 95°C for 10 minutes) and 40 cycles of a two-step PCR (92°C for 15 s, 60°C for 1 minute). The fluorescence intensity of each sample was measured post-PCR in an ABI Prism 7700 sequence detection system (Applied Biosystems), and *SIPA1 *SNP genotypes were determined by the fluorescence ratio of the nucleotide-specific fluorogenic probes.

### Statistical analysis

We used Student's *t* test to compare means for continuous variables and Wilcoxon's sum rank test to compare medians. Variables that were not normally distributed such as tumor size were log transformed. χ^2 ^test or Fisher's exact tests were used to test for differences between categorical variables and testing for Hardy-Weinberg equilibrium. Unconditional logistic regression adjusting for multivariate covariates such as age at diagnosis was used to estimate the adjusted odds ratios (ORs). Lewontin's D prime (D') and correlation coefficient (r^2^) were calculated as two measures of linkage disequlibrium. Using the E-M algorithm [17], we estimated frequencies of the most common haplotypes (with a frequency >1%) in this population and we imputed expected haplotypes for each subject. Haplotype specific ORs were calculated from unconditional logistic regression adjusting for multivariate covariates. We used likelihood ratio tests to calculate *P *values comparing a model with haplotypes versus a model without. All *P *values presented are two tailed and were considered to be statistically significant if they were below 0.05.

## Results

### Patient population

Descriptive data on breast cancer patients included in the study are summarized in Table 2, in which the two case groups are described (patients with localized disease [*n *= 146] and cases patients regional and metastatic disease [*n *= 154]). The data in Table 2 show that the group with localized disease was about 4 years older than those with advanced disease at diagnosis (mean age at diagnosis: 59.7 years versus 55.2 years; *P *= 0.0042). Those with localized disease had a smaller mean tumor size (1.6 cm versus 3.1 cm; *P *< 0.0001). In addition, the group with localized disease had a higher frequency of well differentiated tumors (*P *< 0.0001). No difference in the distribution of estrogen receptor (ER)-positive tumors and a marginal statistical significant difference in progesterone receptor (PR)-positive tumor status were observed between the two study groups. No statistically significant associations were observed between stage of the disease and other epidemiologic risk factors such as alcohol use, smoking, age at menarche, menopause status, parity, estrogen use, and family history of breast, ovarian, or prostate cancer among first-degree relatives.

### Analysis of *SIPA1 *single nucleotide polymorphism genotype frequencies

The allele frequencies for the three SNPs in all breast cancer patients included in the study were 0.359 for SNP -313G>A, 0.556 for SNP 545C>T, and 0.144 for SNP 2760G>A. For none of the SNPs did the distribution deviate from Hardy-Weinberg equilibrium. All three SNPs were in pairwise linkage disequlibrium, as indicated by the parameter D', with D' values of 0.94 between rs931127 and rs3741378, 0.85 between rs931127 and rs746429, and 0.86 between rs3741378 and rs746429.

In a univariate analysis between the distribution of the genotypes and tumor characteristics, we observed the following. First, -313G>A SNP genotypes were significantly associated with the presence of positive lymph nodes at diagnosis; 33.3% of those with GG genotype had positive lymph nodes as compared with 52.7% among those with GA or AA genotype (*P *= 0.0139). Second, 545C>T was associated with ER-negative tumors, with 15.3% of those with CC genotype being ER negative as compared with 34.8% among those with CT or TT genotype (*P *= 0.0006); it was also associated with PR-negative tumors, with 27.8% of those with CC genotype being PR negative as compared with 42.0% among those with CT or TT genotype (*P *= 0.0350). This indicated that the presence of the T allele is associated with a number of indicators of a more aggressive disease process. Third, the frequency distribution of genotypes for the 2760G>A SNP revealed a significant association between the presence of the AA or GA genotype and advanced disease and the presence of positive lymph nodes at diagnosis. The combined frequency of homozygote variant AA and heterozygote GA genotypes was significantly higher in those individuals with nonlocalized, advanced disease than in individuals in whom the tumor remained confined to the breast (65.3% versus 50.4%; *P *= 0.0154). A similar pattern was observed with respect to lymph node involvement, in which the combined frequency of homozygote variants (AA) and heterozygotes (GA) was higher in those individuals with metastasis in one or more axillary lymph nodes than in those with no nodal involvement (64.5% versus 50.4%; *P *= 0.0074). These findings indicate that the variant A allele is associated with a more aggressive disease process.

The genotype frequencies for each of the three SNPs in the study population by tumor characteristics are presented in Table 3. In addition, Table 3 shows the univariate and multivariate-adjusted OR and 95% confidence interval (CI) of the tumor characteristics according to the genotypes of the three SNPs, assuming a dominant model. In the multivariable analysis we included age at diagnosis in all models. Thus, for the -313G>A SNP the multivariate OR of the presence of positive lymph nodes was 2.15 (95% CI 1.14–4.06;*P *= 0.0083). This implies that an individual with the GA or AA genotype was 2.15 times more likely to have positive lymph nodes than was an individual with GG genotype. The multivariable OR of the -545C>T allele and ER and PR status were 2.90 (95% CI 1.52–5.48; *P *= 0.0012) and 1.86 (95% CI 1.05–3.30;*P *= 0.0339), respectively. Similarly, for the -2760G>A SNP the multivariate OR of the presence of positive lymph nodes was 2.05 (95% CI 1.23–3.44;*P *= 0.0062). This implies that an individual with the GA or AA genotype was 2.05 times more likely to have positive lymph nodes than was an individual with the GG genotype.

### Analysis of *SIPA1 *single nucleotide polymorphism haplotype frequencies

We investigated the association between haplotypes defined by these three SNPs and the presence of positive lymph nodes, and ER and PR status. There were a total of seven haplotypes that were imputed from the data on 260 patients with complete genotypes on all three SNPs. However, four of those haplotypes accounted for more than 98% of the observations. Table 4 summarizes the estimated frequencies of the predicted haplotypes, and Table 5 presents the haplotype-specific ORs of the association of haplotypes and the presence of positive lymph nodes, and ER and PR status compared with the most common haplotype (haplotype 1). Haplotypes with allele frequency below 1% were included with the baseline in our analysis. In the analysis of the presence of positive lymph nodes and haplotypes, the haplotype specific OR for haplotype 3 was 2.20 (95% CI 1.27–3.81; *P *= 0.0051) compared with haplotype 1. Thus, women with the estimated haplotype 3 were 2.20 times more likely to present with positive lymph nodes than were women with haplotype 1. In the analysis of ER status and haplotypes, the haplotype specific OR for haplotype 4 was 2.85 (95% CI 1.36–5.94; *P *= 0.0053) compared with haplotype 1. Thus, women with the estimated haplotype 4 were 2.85 times more likely to present with an ER-negative tumor than were women with haplotype 1. In the analysis of PR status and haplotypes, the haplotype-specific OR for haplotype 4 was 2.16 (95% CI 1.13–4.12; *P *= 0.0199) compared with haplotype 1. Thus, women with the estimated haplotype 4 were 2.16 times more likely to present with a PR-negative tumor than were women with haplotype 1.

## Discussion

In this preliminary study we observed strong associations between the three *SIPA1 *SNPs and indicators of aggressive breast cancer in this population. Specifically, we report a number of particularly interesting findings regarding nodal status at the time of diagnosis and tumor sex hormone receptor status; these findings suggest that *SIPA1 *germline variation contributes to metastatic potential in breast cancer. With regard to lymph node involvement, the variant -313G>A and 2760G>A alleles were strongly associated with axillary node involvement at the time of diagnosis on univariate analysis. Indeed, the apparent effect of these SNPs on the frequency of metastasis that was evident upon univariate analysis was complemented by the outcome of multivariate and haplotype frequency estimation. Multivariate analysis of genotype frequencies showed that possession of either of the -313G>A or 2760G>A variant alleles was associated with a significantly increased likelihood of nodal involvement. Similarly, haplotype analyses demonstrated that the haplotype consisting of the wild-type allele of 545C>T and the variant alleles of the two other studied SNPs (haplotype 3; Table 5) is associated with nodal involvement at the time of diagnosis. This apparent association with nodal involvement at the time of diagnosis is particularly interesting given that there was no evidence of association of *SIPA1 *genotype status and tumor size. It was previously demonstrated that a linear relation exists between tumor diameter and the percentage of cases with positive lymph node involvement [18]. One would therefore expect similar associations with tumor size, given the apparent relationship with nodal status at diagnosis. However, this was not the case in the present study, which may well be a consequence of inadequate statistical power to detect such an association. Alternatively, *SIPA1 *polymorphism might facilitate nodal metastasis independent of tumor size. Whichever is the case, further analysis of the significance of these polymorphisms in different populations is required to clarify the situation.

*SIPA1 *polymorphisms in this population were also associated with other features of tumors with aggressive behavior. Positive sex hormone receptor status correlates with favorable prognostic features, including a lower rate of cell proliferation and histological evidence of tumor differentiation [19]. The 545C>T variant allele was associated with ER-negative and PR-negative tumors in this cohort. In addition, haplotype 4, which is defined by the variant allele 545C>T, is strongly associated with ER and PR tumor status (Table 5). This again highlights the deleterious effect that *SIPA1 *germline variation has on prognosis in primary breast carcinoma and implies that variant forms of this gene are associated with more aggressive disease forms.

The importance of these data is heightened when one considers the outcome of our linkage and functional studies of *Sipa1 *in a murine population [12,13,20], coupled with consideration of the known physiologic functions of *SIPA1*. Traditional dogma has stated that cancerous cells gain the ability to metastasize as a consequence of somatic mutation (for example, through loss of heterozygosity or epigenetic events). However, it has become increasingly accepted that hereditary factors influence all stages of tumor pathogenesis in apparently sporadic cancers. These heritable defects will be of low penetrance, but hypothetically they could affect any part of the neoplastic cascade, including modulation of metastatic efficiency. Evaluation of quantitative trait loci in inbred mouse models has proven a particularly powerful means of studying the effects of this type of genetic variation. The present study is an additional example of how mouse quantitative trait locus data, when used in an appropriate manner, can help to unravel the complexities of hereditary influences in sporadic human cancer [21].

Although the specific mechanism by which *SIPA1 *modulates metastatic efficiency is currently unclear, it is worthwhile to speculate on how observations of *SIPA1 *functionality in mice and humans may relate to the process of tumor progression and characterization. Specifically, tumor development in a background of germline-encoded *SIPA1 *dysfunction may enhance the ability of tumor cells to escape the primary lesion through alteration in cell morphology/polarity and/or by weakening the strength of intercellular contacts. This argument gains further potency when one considers the negative regulatory effect of *SIPA1 *on Rap1, and that Rap1 has been implicated in the maintenance of epithelial polarization [22] as well as in the maintenance of intercellular adherens junctions [23]. Further studies will be required to confirm whether this is the case.

*SIPA1 *has been shown to have GTPase activity that is specific for Rap1 and Rap2, both of which are members of the Ras family of GTPases, and are involved in regulating cell proliferation, differentiation, and adhesion [24]. Indeed, *SIPA1 *appears to play a prominent role in regulating cell adhesion, and transient expression of this gene in HeLa cells induces cellular rounding up and detachment from the culture surface [25]. It appears to act by modulating a number of cellular adhesion molecules, for example β_1_-integrin, and thus regulates the interactions between these molecules and other cellular and extracellular matrix proteins involved in the process of adhesion [26-28]. It has been hypothesized that *SIPA1 *negatively regulates integrin-mediated cell adhesion via Rap1 GAP activity [27]. Specifically, *SIPA1 *interacts with the cytoskeletal-anchoring protein AF6 through a common protein-protein interaction motif known as a 'PDZ domain' [27].

In addition to the effects on intercellular adhesion, disruption of *SIPA1 *has been shown to have potent effects on cellular proliferation. For example, *SIPA1*-deficient mice develop a spectrum of symptoms resembling those seen in human myelodysplastic and myeloproliferative disorders [29,30]. This is especially noteworthy because metastatic cells must be able to proliferate effectively at a distant site in what must be considered a foreign microenvironment, if they are to become a clinically relevant metastatic lesion. When all this evidence is considered, we argue that functional data from both mouse and human studies strongly support the findings of the present case-only study, in which we demonstrate that germline polymorphisms and haplotypes of *SIPA1 *are associated with enhanced metastatic efficiency in primary breast carcinoma.

However, further experiments will be required to confirm the role of *SIPA1 *germline variation in modulating tumor progression efficiency, the most obvious of which is to replicate the associations seen here in other populations. It is also vital to resequence various elements of the *SIPA1 *gene to attempt to identify novel polymorphisms, the most critical of which is that encoding the PDZ protein-protein interaction domain – a region that is critical to *SIPA1 *functionality [27]. This endeavor is especially important given that the exon 12 2760G>A SNP – a noncoding, synonymous polymorphism – is strongly associated with aggressive disease in this study. This may well be a reflection of the physical proximity of this SNP to the other polymorphisms characterized in the study, which could have a more plausible effect on *SIPA1 *functionality. This hypothesis is somewhat supported when one considers that a haplotype containing the 2760G>A variant allele is associated with more aggressive disease at diagnosis, as measured by positive lymph node involvement. Conversely, it may well be that the 2760G>A SNP is in linkage disequilibrium with a nearby yet uncharacterized SNP with a more obvious functional effect. Indeed, one could postulate that the associations observed in the present study are in fact a consequence of another gene in linkage disequilibrium with *SIPA1*, because the functional significance (if any) of each of the polymorphisms has not been assessed. However, we argue that this is unlikely because we investigated genes in the chromosomal regions surrounding *Sipa1 *in the mouse and found no data supporting the hypothesis that surrounding genes are involved in modulating metastatic efficiency [13]. This, in combination with observations relating to *Sipa1 *functionality in the mouse and the fact that human haplotype blocks are probably significantly smaller than in inbred mice, leads to the conclusion that the observed associations are a result of *SIPA1 *polymorphism.

The implications of this work are potentially far reaching in that it may well prove possible to assess metastatic potential through genotyping polymorphisms in germline DNA. The ability to evaluate the risk for metastatic disease from readily available tissues such as blood could be a powerful means to augment currently available means of assessing prognosis in breast cancer. However, we do acknowledge that the effect of germline *SIPA1 *variation in isolation is probably too low to allow for the development of an assay that would possess sufficient sensitivity and specificity to determine metastatic propensity accurately. However, it is plausible that a panel of polymorphisms derived from a number of metastasis 'susceptibility' genes would possess the power and accuracy to enable the development of a clinically relevant prognostic assay, which will necessitate the identification of further metastasis efficiency modifier genes.

## Conclusion

Our results imply that *SIPA1 *germline polymorphisms are associated with aggressive disease behavior in the present cohort. If the results of this study hold true in other populations, then the potential for this to have a positive impact on current breast cancer staging and treatment protocols may be great. For example, staging of primary breast cancer in cases in which there is no palpable evidence of nodal metastasis typically requires axillary dissection to assess nodal status. However, controversy exists as to the actual benefit of nodal sampling in those patients with small tumors because of the morbidity frequently associated with this procedure. For example, the incidence of axillary node relapse in patients with stage T1a tumors (tumor >0.1 cm but not >0.5 cm in greatest dimension) treated without axillary dissection was 2% [31]. It is plausible that analysis of *SIPA1 *polymorphisms could be used as an additional element of the staging process that might allow axillary node-negative patients with small primary tumors (for example, T1-2 N0) to be classed as being at increased risk for development of metastasis. In turn, this could then become a powerful influence on whether to initiate adjuvant therapy in patients who would not currently be considered for this type of treatment.

## Abbreviations

CI = confidence interval; D' = Lewontin's D prime; ER = estrogen receptor; NCBI = National Center for Biotechnology Information; OR = odds ratio; PCR = polymerase chain reaction; PR = progesterone receptor; SNP = single nucleotide polymorphism.

## Competing interests

The authors declare that they have no competing interests.

## Authors' contributions

NC was responsible for genotyping polymorphisms, contributed to study design, and drafted the manuscript. The University of California Irvine investigators played several roles in this report. HA-C contributed to the study design, the recruitment of subjects and the interviewing and biospecimen collection, and drafting and editing of the manuscript. AZ contributed to the study design, recruitment of subjects and data analysis, and drafting and editing of the manuscript. DP contributed to study design, biospecimen processing, and drafting and editing of the manuscript. JH participated in data processing and analysis, and editing of the manuscript. KH participated in study design, was responsible for supervising laboratory work, and was involved in manuscript preparation. All authors read and approved the final manuscript.

## Figures and Tables

**Table 1:** PCR primer and SNP-specific fluorogenic oligonucleotide probe sequences

*SIPA1 *polymorphism		Sequence^a^
-313G>A (rs931127)	Forward primer	5'-GCGCGCTAGTGGAATGGTT-3'
	Reverse primer	5'-GACCAACCGGGAGGGCTA-3'
	Wild-type probe	VIC-AGGGCCCAGTGCT
	Variant probe	FAM-AGGGCCCAGTACTTA
545C>T (F182S; rs3741378)	Reverse primer	5'-CCAGCTCGGACCTGCTGCAT-3'
	Reverse primer	5'-GATGGACACGGCCGCGTT-3'
	Wild-type probe	VIC-ACCAGCATCCCCACC
	Variant probe	FAM-ACCAGCATTCCCAC
2760G>A (A920A; rs746429)	Forward primer	5'-CCTTGTGCCCACAGTCATGTC-3'
	Reverse primer	5'-CGGGACAGCGACTCCAGAATG-3'
	Wild-type probe	VIC-CACTGCCCGCCTC
	Variant probe	FAM-CCCACTGCCTGCC

^a^FAM, 5-(&6)-carboxyfluorescein; SNP, single nucleotide polymorphism; VIC, Applied Biosystems^® ^proprietary dye.

**Table 2:** Participant characteristics by localized and regional/metastatic disease

Characteristic		Localized (*n *= 148)	Regional/metastatic (*n *= 152)	*P*
Age at diagnosis	<50 years	44 (30.1%)	57 (37.0%)	0.0042^a^
	≥50 years	102 (69.9%)	97 (63.0%)	
	Mean age (years; mean ± SD)	59.7 ± 14.3	55.2 ± 12.6	
	Range (years)	33–91	25–87	
ER status	Positive	109 (74.7%)	105 (68.2%)	0.1741^b^
	Negative	22 (15.0%)	33 (21.4%)	
	Unknown	15 (10.3%)	16 (10.4%)	
PR status	Positive	96 (65.7%)	87 (56.5%)	0.0489^b^
	Negative	33 (22.7%)	451 (33.1%)	
	Unknown	17 (11.6%)	16 (10.4%)	
Tumor size	< 2 cm	102 (69.9%)	55 (35.7%)	<0.0001^c^
	≥ 2 cm	34 (27.4%)	84 (54.6%)	
	Unknown	4 (2.7%)	15 (9.7%)	
	Mean size (cm; mean ± SD)	1.6 ± 1.0	3.1 ± 2.7	
	Range (cm)	0.1–6.5	0.4–18.0	
Grade	Well to moderate	86 (58.9%)	46 (29.9%)	<0.0001^b^
	Poor	26 (17.8%)	79 (51.3%)	
	Unknown	34 (23.3%)	29 (18.8%)	

Values are expressed as *n *(%), unless otherwise stated. ^a^Based on a t-test. ^b^Based on a Fisher's exact test excluding the missing values. ^c^Based on a t-test on the logarithmic scale. ER, estrogen receptor; PR, progesterone receptor; SD, standard deviation.

**Table 3:** Genotype distribution by tumor characteristics

SNP genotype	Disease characteristic (*n *[%])	Odds ratios		95% CI	*P*
				
		Crude	Adjusted^a^		
-313G>A (rs931127; negative/positive nodes)					
GG	36 (27.5)/18 (14.5)	1.00	1.00		
GA	58 (44.3)/60 (48.4)	2.23	2.15	1.14–4.06	0.0083
AA	34 (28.2)/46 (37.1)				
545C>T (rs3741378; ER positive/ER negative)					
CC	166 (78.7)/30 (55.6)	1.00	1.00		
CT	39 (18.5)/22 (40.7)	2.95	2.90	1.52–5.48	0.0012
TT	6 (2.8)/2 (2.0)				
545C>T (rs3741378; PR positive/PR negative)					
CC	140 (77.8)/54 (65.1)	1.00	1.00		
CT	35 (19.4)/26 (31.3)	1.88	1.86	1.05–3.30	0.0339
TT	5 (2.8)/3 (3.6)				
2760G>A (rs746429; negative/positive nodes)					
GG	64 (49.6)/41 (32.5)	1.00	1.00		
GA	46 (35.7)/65 (51.6)	2.04	2.05	1.23–3.44	0.0062
AA	19 (14.7)/20 (20.0)				

^a^Adjusted analysis models include age at diagnosis. CI confidence interval; ER, estrogen receptor; progesterone receptor.

**Table 4:** Estimated frequencies of predicted haplotypes

Haplotype	-313G>A (rs931127)	545C>T (rs3741378)	2760G>A (rs746429)	Estimated frequency (%)
1	G	C	G	42.1
2	A	C	G	8.0
3	A	C	A	34. 6
4	A	T	G	13. 5
5^a^	G	C	A	0.9
5^a^	G	T	G	0.5
5^a^	A	T	A	0.3

Haplotypes frequencies were estimated from the 260 samples with complete genotype data on all three polymorphisms. ^a^Haplotypes with an estimated frequency below 1% were pooled for the purposes of analysis.

**Table 5:** Haplotype specific odds ratios adjusted for age and association with tumor characteristics

Haplotype^a^	Positive nodes		ER status		PR status	
	
	OR (95% CI)	*P*	OR (95% CI)	*P*	OR (95% CI)	*P*
1	1.00		1.00		1.00	
2	1.14 (0.52–2.51)	0.7410	0.93 (0.34–2.60)	0.8961	0.99 (0.42–2.33)	0.9828
3	2.20 (1.27–3.81)	0.0051*	0.62 (0.30–1.29)	0.2005	1.50 (0.82–2.77)	0.1915
4	1.00 (0.54–1.86)	0.9940	2.85 (1.36–5.94)	0.0053*	2.16 (1.13–4.12	0.0199*

^a^Haplotypes with an estimated frequency under 1% were pooled within the baseline for the purposes of analysis. *Statistically significant comparisons of haplotype frequencies.
